# HIV/aids related home based care practices among primary health care workers in Ogun state, Nigeria

**DOI:** 10.1186/1472-6963-12-112

**Published:** 2012-05-07

**Authors:** Amoran E Olorunfemi, Elijah O Ogunsola, Albert O Salako, Ok O Alausa

**Affiliations:** 1 Department of Community Medicine and Primary Care, College of Health Sciences, Olabisi Onabanjo University Teaching Hospital, Sagamu, Nigeria; 2 Department of Community Medicine and Primary Care, Olabisi Onabanjo University Teaching Hospital, Sagamu, Nigeria

## Abstract

**Background:**

HIV/AIDS is fast becoming a chronic disease with the advent of antiretroviral drugs, therefore making home based care key in the management of chronically ill HIV/AIDS patient. The objective of this study was to determine the perception and practice of health care workers on HIV/AIDS related home based care in the health facilities in Ogun state, Nigeria.

**Methods:**

This study is an analytical cross-sectional study. A multistage cluster sampling technique was used to obtain a representative sample of the primary health care workers in Ogun state. An interviewer administered structured questionnaire was administered by trained health workers to elicit the required information.

**Result:**

A total of 350 health care workers were interviewed, 70% of the respondents could adequately describe the components of home based care. Only 38.7% were aware of the National guideline on home based care practices and 17.1% believe that home based care will not significantly improve the prognosis of PLWAs. Few 19.1% had ever been trained or ever involved 16.6% in home based care practices. Only 20 [5.7%] are involved on a weekly basis, 16 [4.6%] monthly and 22 [6.3%] quarterly. Reasons given for non implementation of home based care are inadequate number of healthcare workers 45%, lack of political will 24.4%, lack of implementation by facility managers 14% and inadequate funds 16.6%.

Factors that were significantly associated with the practice of home based care were perception of its relevance in improving prognosis [OR = 54.21, C.I = 23.22-129.52] and presence of a support group in the facility [OR = 4.80, C.I = 2.40-9.57]. There was however no statistically significant relationship between adequate knowledge of home based care [OR = 0.78, C.I = 0.39-1.54] and previous training on home based care (OR = 1.43, C.I = 0.66-3.06].

**Conclusion:**

The practice of home based care for HIV/AIDS among the study population is low and it is greatly influenced by perception of its effectiveness and relevance. The study recommends that the health care workers should be adequately educated on the importance of home based care in the management of chronic illnesses in order to enhance its practice.

## Background

Living and dying with incurable illness in poverty and pain is all too common in sub-Saharan Africa [1,2]. With minimal resources and huge shortages of health workers, national health systems in a number of African countries have focussed primarily on preventive, curative and maternal health services thus responding to a set of immediate health problems [1]. In many countries, minimal resources have been dedicated to supportive or palliative care [3]. However, the severity of the HIV and AIDS pandemic has triggered donor interest in palliative and home based care in Africa, an interest which has never been greater, as evidenced by multiple small projects [3,4] and promotion by various national and pan-African initiatives [5,6].

There is ample evidence that home based care services and practice is prominent especially among informal health care workers such as traditional birth attendants [TBAs], Voluntary health care workers etc. in Nigeria though the practice among the formal health care workers is rare [7-10]. This is done in spite of a relatively easy access to institutional maternity services in urban areas. Previous studies about home health care in urban and peri-urban areas have reported that the utilization of home based services is more closely related to the needs of caregivers than the care needs level of the users [2]. It is usually determined by multidimensional services indicators and care needs such as poor education, multiparity, and low socioeconomic status in rural areas in Africa [4,11-13]. Although there have been a few number of studies that focused on care at home, more information is needed about the range and severity of problems encountered by primary care professionals and the barriers that prevent optimal care in order to identify the educational and training needs of primary care teams.

The World Health Assembly in 2005 even declared supportive or palliative care as an urgent humanitarian responsibility [14]. Despite various palliative care programmes which have been developed, it is still only available to less than 5% of those in Africa who need it [14]. Assessment of the perception and practice of home based care by health care workers is vital to understanding the extent of practice among the study population and also to determine the factors that encourage its practice in an African population. This study was therefore designed to determine the perception and practice of health care workers on HIV/AIDS related home based care in the health facilities in Ogun state, Nigeria.

## Methods

### Study design

This study is an analytical cross-sectional study. The information was collected from health care workers in Abeokuta South local government area of Ogun State from February 10, 2008 to April 15, 2008.

### The study area

The study was conducted in Ogun State which is one of the 36 states in Nigeria and was created in February 1976 out of the old western state. It is located in the South West Zone (which is one of the 6 geo-political zones) of Nigeria with a total land area of 16,409.26 square kilometers and a population of 3,507,735 with growth rate of 2.83% per annum (2006 National Census). However, the estimated HIV/AIDS prevalence in Ogun state was 1.7% in 2008 [15]. The state has twenty (20) Local Government Areas, with each LGA headed by an executive chairman. It is divided into 3 senatorial districts and 26 state constituencies.

This study was conducted in Abeokuta South local government area of Ogun State from February 10, 2008 to April 15, 2008. All health workers in primary health care facilities who consented to take part in the study were recruited.

### Sampling technique

A multistage cluster sampling technique was used to obtain a representative sample of all the primary health care workers in Ogun state.

Stage 1: The framework of all the local government in Ogun state was obtained from the National population commission. Abeokuta South local government area local government area was selected by balloting among all the 20 local government areas in Ogun state.

Stage 2: All health care workers in primary health care facilities in Abeokuta South local government area of Ogun State from February 10, 2008 to April 15, 2008 who consented to take part were enrolled in the study.

### Sampling size determination

The sample size was calculated using Epi-Info version 6.0 statistical software. The result of a previous study [4] that showed 17% prevalence of home care delivery was used.

The sample size used for this study was calculated with the formula

(1) $$\begin{array}{l} n=z^{2}\text{pq}/d^{2}\\ \text{n }=\;1.96^{2}\times 0.17\times 0.83\\ 0.05^{2}=217 \end{array}$$

However a total of Three hundred and fifty participants [350] were recruited into the study.

### Study instrument

The factors examined in the study include socio-demographic characteristics (age, sex, income, education, religion, marital status, occupation, place of residence) and the knowledge of components of HIV/AIDS related home based care and factors associated with the practice of home based care.

Single multiple response choice questions were asked to determine the practice of home based care.

The Questionnaire was pre-tested on health workers in the Reproductive health unit of the Ogun State Ministry of Health, and necessary adjustments were made. Trained data collectors explained the aim of the study, obtained informed consent, and interviewed each respondent privately. All information was obtained under anonymity and the data was collected by trained personnel on clinic days.

### Ethical considerations

Ethical clearance: was obtained from the Ogun State Ministry of Health Ethics Board. Confidentiality on candidate's information was maintained. Permission of the State Ministry of Health, HIV/AIDS Control Division were obtained before the commencement of the study.

### Data analysis

To describe patient socio-dermographic characteristics, we calculated proportions and medians. For categorical variables, we compared proportions using chi-square tests and, when appropriate, Fisher's exact test. For continuous variables, we compared medians using the Wilcoxon Rank-Sum Test. Chi-square was used to determine association between categorical variables and a p value of less than 0.05 was considered significant. The relationships between socio-demographic characteristics of the health care workers and their practice of Home based care services were examined through bivariate analysis, by computing odds ratio at 95% confidence level and chi square and t-tests where appropriate. Data were presented in tabular form.

## Results

### Socio-dermographic characteristics of respondents

A total of 350 health care workers were recruited into the study. The age of the respondents ranged from 21 to 59 years, (mean 39.42 ± 7.15 years). Majority [64.9%] of the respondents were married and 30.9% were never married. Most of the respondents 286 [81.7%] were Christians, 17.4% Muslims and 3[0.9%] were traditional worshippers. Three quarters 75.7% of the respondents were females. More than half 218 [63.6%] of the respondents were from the nursing profession, 50 [14.6%] were community health workers, 6.9% were Medical doctors and 58 [14.9%] were from other paramedical professions such as Laboratory scientists, Pharmacists, Medical records etc. Majority [71.4%] have been working for between 11-20 yrs, 80 [22.5] for less than 10 yrs and 20 [5.7%] for more than 20 yrs while 285 [%] have been working in public health field since employment. The prevalence of practice of home based care among these health workers was 58 [16.6%] (Table 1).

**Table 1:** The Socio-dermographic characteristics of respondents

	**No of respondents**	**% of respondents**
**Age**		
21-30 yrs	93	27.3
31-40 yrs	124	36.4
41-50 yrs	39	27.3
51-60 yrs	40	9.0
Total	350	100.0
**Sex**		
Male	85	24.3
Female	265	75.7
**Marital Status**		
Never married	108	30.9
Married	227	64.9
Seperated/Divorced	6	1.7
Widow/Widower	9	2.5
**Profession**		
Medical doctors	24	6.9
Nurses	218	63.6
Community Health Workers	50	14.6
Others	58	14.9
**Religion**		
Christainity	286	81.7
Islam	61	17.4
Traditional Religion	3	0.9
**Years of working experience**		
0-10 yrs	80	22.8
11-20 yrs	250	71.4
>20 yrs	20	5.7
**Working in Public Health field**		
Yes	285	81.4
No	65	18.6

### Perception of the health care workers about home based care

Two hundred and fifty [70%] of the respondents had adequate knowledge of all the elements of HIV/AIDS related home based care. 60 [17.1%] believed that home based care is not essential or relevant to the management of HIV/AIDS in their environment. Only 67 [19.1%] of the respondents have ever received any form of training on home based care. Only those 58 [16.6%] that practice home based care believed that its practice is feasible while 150 [42.9%] believed that it is an expensive venture and 142 [40.1%] believed that it is a cumbersome practice. Majority [55/58] of the respondents that practice home based care have support group in their facility. Among those that practice home based care only 16 [5.7%] do home visit weekly, 4.6% monthly and 6.3% on a quarterly basis. Reasons given for non implementation of home based care are inadequate number of healthcare workers 45%, lack of political will 24.4%, lack of implementation by facility managers 14% and inadequate funds 16.6%.

Table 2 shows the perception and practice of home based care among the respondents.

**Table 2:** Knowledge, perception and practice of home based care among HCW

	**No of respondents**	**% of respondents**
**Knowledge of home based care**		
Adequate	245	70.0
Inadequate	105	30.0
**Relevance of Home based care**		
Relevant	290	82.9
Not relevant	60	17.1
**Trained on HBC**		
Trained	67	19.1
Not trained	283	80.9
**Practice HBC**		
Weekly	20	5.7
Monthly	16	4.6
Quarterly	22	6.3
Nil Practice	292	83.4
**Support group in your facility**		
Yes	55	15.7
No	295	84.3
**Opinion about HBC**		
Feasible	58	16.6
Expensive	150	42.9
Cumbersome	142	40.5

### Factors associated with the practice of HIV/AIDS related home based care

The practice of home based care was statistically significantly associated with perception of its relevance in the management of HIV/AIDS. Those who believe that it significantly improves prognosis reported home visits [OR = 54.21, C.I = 23.22-129.52]. More of those that have ever been trained for home based care practice it (OR = 1.43, C.I = 0.66-3.06] but it was not statistically significantly related to practice. Those from the facilities that have support group were about 5 times more likely to report the practice of home based care when compared to those from facilities that do not have support group [OR = 4.80, C.I = 2.40-9.57]. There was however no statistically significant relationship between adequate knowledge of home based care and its practice [OR = 0.78, C.I = 0.39-1.54] Table 3 shows the influence of knowledge and perception of home based care on its practice.

**Table 3:** Factors associated with practice of HBC services

	**Total**	**Practices HBC**	**Do not practice HBC**	***X***^**2**^**[p-value]**	**Unadjusted odds ratio**
**Knowledge of HBC**					
Adequate	245 [70.0]	43	202	0.5	0.78 [0.4-1.5]
				[0.5]	
Inadequate	105 [30.0]	15	90		1.00
Total	350 [100.0]	58	292		
**Perception about HBC**					
Relevant	60 [17.1]	44	16	168.3	54.21 [23.2-129.5]
				[<0.001]	
Not relevant	290 [82.9]	14	276		1.00
**Training**					
Trained	67 [19.1]	12	45	1.0 [0.3]	1.4 [0.7-3.1]
Not trained	283 [80.9]	46	247		1.00
**Availability of support group in facility**					
Yes	55 [15.7]	22	33	25.8	4.8 [2.4-9.6]
				[<0.001]	
**No**	295 [84.3]	36	259		1.00

## Discussion

The study shows that the prevalence of practice of home based care among these public health workers was low [16.6%]. The fact that home based care practices among health care workers was low has been widely reported by several studies especially in low socioeconomic settlements [7,11-13]. The practice of home based care among the study population was significantly associated with perception of its relevance in the management of HIV/AIDS. The study shows the need for community-based interventions to promote improved home based care practices in urban and rural areas. Implementation of an effective program for promotion of home based care practices requires understanding of the community and household traditional care practices. Such information will enable the development of programs to promote culturally sensitive and acceptable changes in practices.

The effectiveness of home care services on recipients is still controversial. It has been difficult to assure the quality of care research [16], and consequently there is inconsistent evidence in support of the effectiveness of home care services [17]. As a result, some studies have reported positive effects of home care services including reductions in functional decline [18-22], mortality rates [23-27], institutionalization rates [28-30] and costs of care [31]. Other studies, however, have reported that home care services are ineffective in reducing functional decline [20], mortality rates [17,19] and institutionalization rates [31,32] in community-dwelling elderly persons.

Only those that practice home based care believed that its practice is feasible while others believed that it is either an expensive venture or a cumbersome practice. This indicates that the practice of home based care is greatly influenced by cost. Several studies have reported similar findings [9,10] on issues pertaining to the development of home treatment services and on macro-level outcomes such as cost-effectiveness and admission rates, which have political, economic, and practical implications. However, there is a paucity of research regarding the structures and processes in actual operations over time, as well as a lack of research of service-user experiences and satisfaction on key areas of service provision [20,24,33]. Establishing performance indicators for assessing home based care is essential in identifying the effect of home care services and would contribute to standardization and improvement in the quality of home care services. This may be one strategy for addressing the shortage of resources and encouraging health care workers to practice quality home based care [1,3].

The study implies that neither the adequate knowledge of home based care nor its training significantly influenced its practice but those from the facilities that had support group were about 5 times more likely to practice home based care for people living with HIV/AIDS. This shows that practical Information about the reasons for home based care is more important than theoretical knowledge. This may indicate a need for health care planners to design appropriate health care services especially in management of chronic diseases. Several studies have reported similar findings [24-26]. More information is needed about the range and severity of problems encountered by primary care professionals and the barriers that prevent optimal care in order to identify the educational and training needs of primary care teams.

Our study has certain limitations. The study findings are limited in terms of overall generalization and impact since the study might also have been faced with recall bias and it is not all health workers in Abeokuta south local government area that was recruited into the study. Despite these limitations, we believe that our data provide useful information for the assessment and implementation of home based care services in Nigeria and will also inform policy decision in Nigeria and other low income countries.

## Conclusion

The study concludes that the practice of home based care for people living with HIV/AIDS among the study population is low and it is greatly influenced by cost and perception of its effectiveness and relevance. The study recommends that the health care workers should be adequately trained on the practice of home based care and its implementation in order to enhance its practice.

## Competing Interests

The authors declare that they have no competing interests.

## Authors’ contributions

AOE participated in the study design, conducted data collection and prepared the final manuscript. OEA conceived the study theme, participated in the study design and supervised data collection. CEA and AOK were involved in Data collection and analysis. All authors read and approved the final manuscript.

## Pre-publication history

The pre-publication history for this paper can be accessed here:

http://www.biomedcentral.com/1472-6963/12/112/prepub

## References

[B1] Grant E Murray SA Grant A Brown J A good death in rural Africa? Listening to patients and their families talk about care needs at the end of life J Pall Care 2003 19 3159 3167 14606327

[B2] Murray SA Grant E Kendall M Dying from cancer in developed and developing countries: lessons from two qualitative interview studies of patients and their careers BMJ 2003 326 368 10.1136/bmj.326.7385.368 12586671 PMC148895

[B3] Clark D Wright M Hunt J Lynch T Hospice and palliative care development in Africa: a multi-method review of services and experiences J Pain Symptom Manage 2007 33 698 710 10.1016/j.jpainsymman.2006.09.033 17531910

[B4] UNAIDS AIDS epidemic update 2007 2007 WHO, Geneva

[B5] Harding R Stewart K Marconi K O'Neill JF Higginson IJ Current HIV/AIDS end-of-life care in Sub-Saharan Africa: a survey of models, services, challenges and priorities BMC Publ Health 2003 3 33 http://www.biomedcentral.com/1471-2458/3/33 10.1186/1471-2458-3-33 PMC280683 14572317

[B6] Mpanga Sebuyira L Mwangi-Powell F Pereira J Spences C The Cape Town Pallaitive Care Declaration: Home grown solutions for sub-Saharan Africa J Palliat Care 2003 6 341 343 10.1089/109662103322144646 14509478

[B7] Ogunniyi SO Faleyin BL Makinde ON Adejuyigbe EA Ogunniyi FA Owolabi AT Delivery care services utilization in an urban population Niger J Med 2000 9 3 81 85

[B8] Olowonyo MT Adekambi MA Obasanjo-Bello I Findings on the use of antenatal facilities in Ogun State, Nigeria Niger Med Pract 2004 45 68 71

[B9] Dawodu A Neonatology in developing countries: problems, practices and prospects Ann Trop Paediatr 1998 18 573 579 10.1080/02724936.1998.11747984 9876272

[B10] Lamina MA Sule-Odu AO Jagun OE Factors militating against delivery amongst patients booked in OOUTH, Sagamu Niger J Med 2004 13 1 52 55 15296109

[B11] Ayoola AA Orimadegun AF Akinsola AK Osinusi K A five-year review of childhood mortality at the UCH, Ibadan W Afr J Med 2005 24 2 175 179 10.4314/wajm.v24i2.28192 16092323

[B12] Fikree FF Ali TS Durocher JM Rahbar MH Newborn care practices in low socioeconomic settlements of Karachi, Pakistan Soc Sci Med 2005 60 5 911 921 10.1016/j.socscimed.2004.06.034 15589663

[B13] Chowdhury ME Ronsmans C Killewo J Equity in use of home-based or facility-based skilled obstetric care in rural Bangladesh: an observational study Lancet 2006 367 9507 327 332 10.1016/S0140-6736(06)68070-7 16443040

[B14] World Health Assembly Cancer Prevention and Control Resolution 2005 WHA, 22

[B15] Federal Ministry of Health Department of Public Health National AIDS/STDs Control Program. Technical Report 2008

[B16] Harwood RH Ebrahim S Long-term institutional residents: does the environment affect outcomes? J R Coll Phys Lond 1992 26 2 134 138 PMC5375526 1534123

[B17] Bouman A van Rossum E Evers S Ambergen T Kempen G Knipschild P Effects on health care use and associated cost of a home visiting program for older people with poor health status: a randomized clinical trial in the Netherlands J Gerontol A Biol Sci Med Sci 2008 63 3 291 297 10.1093/gerona/63.3.291 18375878

[B18] Gitlin LN Hauck WW Winter L Dennis MP Schulz R Effect of an in-home occupational and physical therapy intervention on reducing mortality in functionally vulnerable older people: preliminary findings J Am Geriatr Soc 2006 54 6 950 955 10.1111/j.1532-5415.2006.00733.x 16776791

[B19] Hughes SL Conrad KJ Manheim LM Edelman PL Impact of long-term home care on mortality, functional status, and unmet needs Health Serv Res 1988 23 2 269 294 3133324 PMC1065503

[B20] Stuck AE Egger M Hammer A Minder CE Beck JC Home visits to prevent nursing home admission and functional decline in elderly people: systematic review and meta-regression analysis JAMA 2002 287 8 1022 1028 10.1001/jama.287.8.1022 11866651

[B21] Huss A Stuck AE Rubenstein LZ Egger M Clough-Gorr KM Multidimensional preventive home visit programs for community-dwelling older adults: a systematic review and meta-analysis of randomized controlled trials J Gerontol A Biol Sci Med Sci 2008 63 3 298 307 10.1093/gerona/63.3.298 18375879

[B22] Beswick AD Rees K Dieppe P Ayis S Gooberman-Hill R Horwood J Ebrahim S Complex interventions to improve physical function and maintain independent living in elderly people: a systematic review and meta-analysis Lancet 2008 371 9614 725 735 10.1016/S0140-6736(08)60342-6 18313501 PMC2262920

[B23] Kono A Kanagawa K Ban M Kitahama Y Matubara E [Evaluation of a community-based preventive care program for elderly living at home] Nippon Koshu Eisei Zasshi 2002 49 9 983 991 12402476

[B24] Elkan R Kendrick D Dewey M Hewitt M Robinson J Blair M Williams D Brummell K Effectiveness of home based support for older people: systematic review and meta-analysis BMJ 2001 323 7315 719 725 10.1136/bmj.323.7315.719 11576978 PMC56889

[B25] Pathy MS Bayer A Harding K Dibble A Randomised trial of case finding and surveillance of elderly people at home Lancet 1992 340 8824 890 893 10.1016/0140-6736(92)93294-W 1357306

[B26] Melis RJ van Eijken MI Teerenstra S van Achterberg T Parker SG Borm GF van de Lisdonk EH Wensing M Rikkert MG A randomized study of a multidisciplinary program to intervene on geriatric syndromes in vulnerable older people who live at home (Dutch EASYcare Study) J Gerontol A Biol Sci Med Sci 2008 63 3 283 290 10.1093/gerona/63.3.283 18375877

[B27] Kuzuya M Masuda Y Hirakawa Y Iwata M Enoki H Hasegawa J Iguchi A Day care service use is associated with lower mortality in community-dwelling frail older people J Am Geriatr Soc 2006 54 9 1364 1371 10.1111/j.1532-5415.2006.00860.x 16970643

[B28] Vass M Avlund K Hendriksen C Andersen CK Keiding N Preventive home visits to older people in Denmark: methodology of a randomized controlled study Aging Clin Exp Res 2002 14 6 509 515 12674492 10.1007/BF03327352

[B29] Landi F Gambassi G Pola R Tabaccanti S Cavinato T Carbonin PU Bernabei R Impact of integrated home care services on hospital use J Am Geriatr Soc 1999 47 12 1430 1434 10591237 10.1111/j.1532-5415.1999.tb01562.x

[B30] Vass M Avlund K Hendriksen C Randomized intervention trial on preventive home visits to older people: baseline and follow-up characteristics of participants and non-participants Scand J Publ Health 2007 35 4 410 417 10.1080/14034940601160763 17786805

[B31] Weissert WG Lesnick T Musliner M Foley KA Cost savings from home and community-based services: Arizona's capitated Medicaid long-term care program J Health Polit Policy Law 1997 22 6 1329 1357 9459131 10.1215/03616878-22-6-1329

[B32] van Haastregt JC Diederiks JP van Rossum E de Witte LP Crebolder HF Effects of preventive home visits to elderly people living in the community: systematic review BMJ 2000 320 7237 754 758 10.1136/bmj.320.7237.754 10720360 PMC27318

[B33] National Audit Office Helping people through mental health crisis: The role of crisis resolution and home treatment services 2007 The Stationery Office, London

